# High-throughput X-ray total scattering measurement system at BL04B2 of SPring-8

**DOI:** 10.1107/S1600577525011294

**Published:** 2026-01-22

**Authors:** Hiroki Yamada, Seiya Shimono, Shogo Kawaguchi, Koji Ohara, Kei Watanabe, Michitaka Takemoto, Jo-chi Tseng, Masakuni Takahashi, Yuki Sada, Hiroshi Yamazaki, Haruhiko Ohashi, Yasuhiko Imai, Takaki Hatsui, Ichiro Inoue, Shigeru Kimura, Kunihisa Sugimoto, Makina Yabashi, Osami Sakata, Yuji Higo, Kenji Tamasaku

**Affiliations:** a Japan Synchrotron Radiation Research Institute 1-1-1 Kouto Sayo-cho, Sayo-gun Hyogo679-5198 Japan; b Faculty of Materials for Energy Shimane University 1060 Nishi-Kawatsu-cho Matsue Shimane690-8504 Japan; c Graduate School of Environmental, Life, Natural Science and Technology Okayama University 3-1-1 Tsushima-naka Kita-ku Okayama700-853 Japan; d RIKEN SPring-8 Center, 1-1-1 Kouto, Sayo-cho, Sayo-gun, Hyogo679-5148, Japan; e Faculty of Science and Engineering, Graduate School of Science and Engineering Kindai University 3-4-1 Kowakae Higashiosaka Osaka577-8502 Japan; Bhabha Atomic Research Centre, India

**Keywords:** pair distribution function, beamline BL04B2, high energy X-ray total scattering, high-throughput measurement, synchrotron radiation, molecular dynamics, structural analysis

## Abstract

X-ray total scattering is widely used for analyzing pair distribution functions (PDFs) to elucidate the local structure of materials. For achieving high-throughput PDF analysis, a beamline monochromator and an experimental system were updated at BL04B2 of SPring-8. The system consists of a diffractometer and 2D area detectors, which cover a wide *Q* range of 0.2–27 Å^−1^ at a photon energy of 113 keV, with a temperature control capability between 100 and 1100 K. The typical measurement time is reduced to ∼10 min per sample, which is more than ten times faster than the conventional setup. This system enables efficient structural analysis of various types of materials such as liquids, glasses and nanocrystals while yielding abundant measurement data for use in materials science.

## Abstract 2

A high-throughput X-ray total scattering system was newly installed in BL04B2, achieving more than ten times faster acquisition times than those of the conventional setup.

## 1. Introduction

The pair distribution function (PDF) (Billinge, 2008 ▸) expresses the probability of existence of other atoms at a specific distance from the center of a reference atom. By evaluating the PDF, the characteristics of local structures from short to medium range (approximately several nanometres) can be elucidated even in glasses and liquids (Sheng *et al.*, 2006 ▸), which lack the periodic order observed in crystals. In recent years, crystalline materials have also increasingly been evaluated by PDF to analyze the structural features such as local distortions. PDF is also widely used for atomic simulations performed using methods such as molecular dynamics (Skinner *et al.*, 2012 ▸).

The PDF can be calculated via simulation or from experiments based on the scattering patterns of high-energy X-rays from synchrotron radiation and/or neutron beams (Soper & Barney, 2011 ▸). By removing the background and incoherent scattering, the structure factor *S*(*Q*) can be obtained from the X-ray total scattering pattern. Subsequently, reduced PDF, *G*(*r*), one of the representations of PDF, is calculated using the following equation,
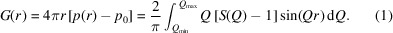
The PDF in equation (1) was derived using a Fourier transform. Theoretically, a PDF with ideal resolution should be obtained if *Q*_max_ can be measured to infinity; however, the measurable *Q*_max_ is limited by the experimental setup, particularly the photon beam wavelength (Egami & Billinge, 2003 ▸). Therefore, high-energy synchrotron light sources are widely used for obtaining high-resolution PDFs with high statistical accuracy and wide *Q*-range. For instance, beamlines such as P02.1 at PETRA III (Dippel *et al.*, 2015 ▸), ID-22 in ESRF (Fitch *et al.*, 2023 ▸), 11-ID-B at APS (Chupas *et al.*, 2007 ▸), 28-ID at NSLS II (Palomino *et al.*, 2017 ▸) and I15-1 at the Diamond Light Source (Sutter *et al.*, 2016 ▸) are used for X-ray PDF analysis with photon energies between 60 and 117 keV, allowing the acquisition of X-ray scattering patterns over a wide *Q* range exceeding 20 Å^−1^. These beamlines are also equipped with large-area flat-panel detectors which enable the rapid measurement of X-ray total scattering patterns.

The beamline BL04B2 at SPring-8 was originally developed for the PDF analysis of amorphous and liquid materials (Kohara *et al.*, 2007 ▸; Kohara *et al.*, 2014 ▸). The measurement system consisted of slits and point detectors to reduce background scattering and detect weak scattering from samples such as water and alcohol. Since this measurement system uses semiconductor-type detectors (CdTe and Ge), fluorescence signals from samples containing heavy atoms can be suppressed by tuning the X-ray energy within the optimal range for obtaining accurate X-ray total scattering patterns (Ohara *et al.*, 2020 ▸). However, the throughput was rather low with a typical measurement time of more than 2–3 h for each sample. For achieving higher throughput, a system with 2D detectors under low-background scattering conditions was required.

To this end, we recently refurbished the beamline monochromator and experimental system of BL04B2. As a result, efficient PDF measurements with the measurable *Q* range 0.2–27 Å^−1^ can now be obtained with a typical acquisition time of 10 min per sample, which is more than ten times faster than the conventional setup on the same beamline. The new configuration enables structural analysis of materials such as glasses, nanocrystals and porous materials, and supports large-volume data acquisition for materials science.

## 2. Development of the apparatus and configuration

### 2.1. Setup and modification of BL04B2

**Figure 1:**
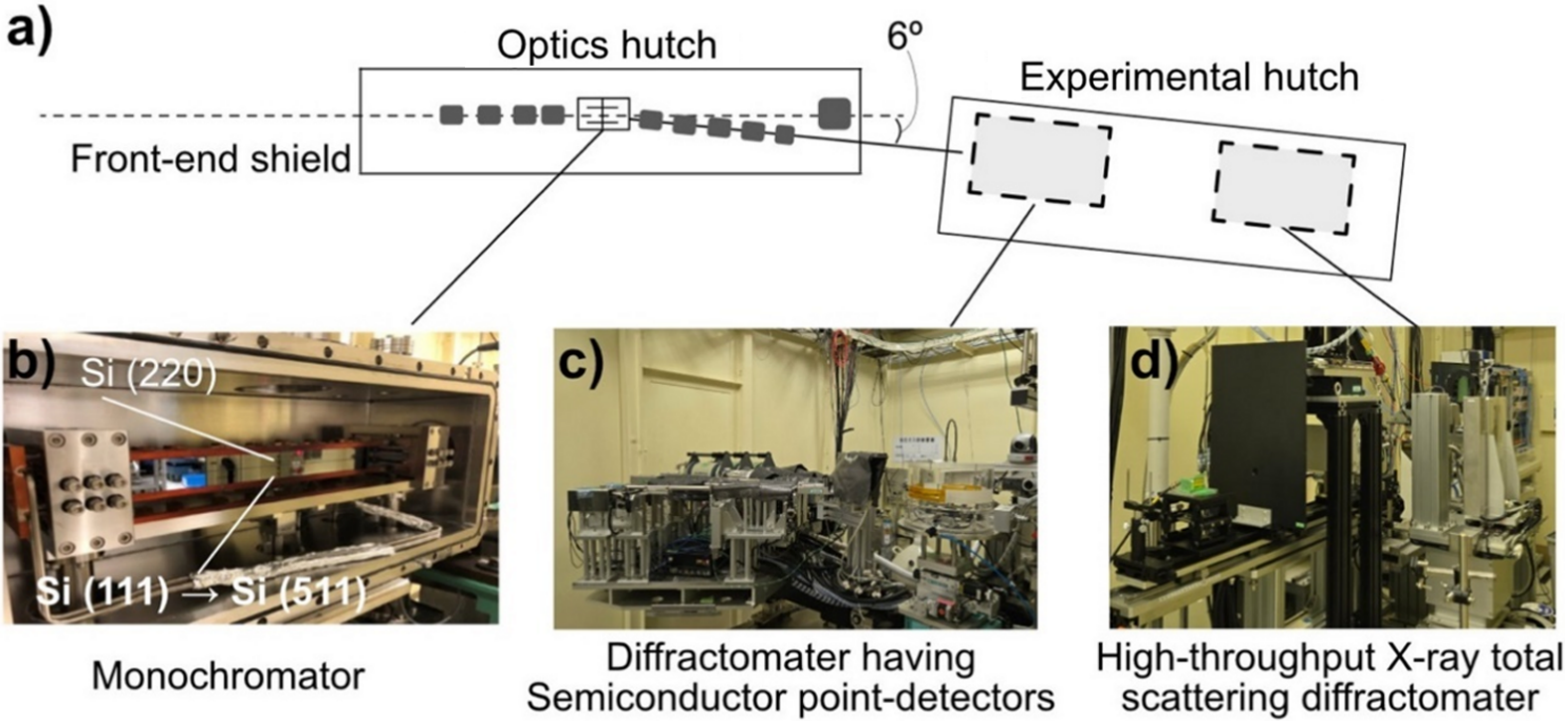
(*a*) Annotated schematic of the optical and experimental hutches at BL04B2. (*b*) Photographs of the Si(220) monochromator designed for X-rays with an energy of 61 keV, and the Si(511) monochromator that replaced the Si(111) monochromator to accommodate X-rays with an energy of 113 keV. (*c*) A two-axis horizontal diffractometer equipped with seven point-type semiconductor detectors was installed upstream. (*d*) A newly developed high-throughput X-ray total scattering diffractometer was implemented in the experimental hutch downstream.

Fig. 1 ▸ schematizes the optics and experimental hutch of the beamline BL04B2 in SPring-8. A bending magnet is used as the X-ray source. Single-bounce bent-crystal monochromators are installed with a fixed Bragg angle of 3°. The monochromatic beam is collimated and shaped using a four-blade slit system located in the optics hutch. The collimated beam is then guided into the experimental hutch, as shown in Fig. 1 ▸(*a*).

A conventional X-ray total scattering diffractometer is located in the upstream region of the experimental hutch [Fig. 1 ▸(*c*)]. This system comprised a combination of point-type semiconductor detectors, as previously described by Yamada *et al.* (2022 ▸). In the present update, we installed the high-throughput X-ray total scattering diffractometer with 2D area detectors downstream of the experimental hutch [Fig. 1 ▸(*d*)].

For the beamline monochromator, an Si(111) crystal or Si(220) crystal was switched in the previous setup, while contamination of multiple harmonics was problematic, particularly for the use of 2D detectors that could not discriminate photon energies. For example, 37.8 keV photons with an Si(111) reflection were contaminated during the use of 113 keV with an Si(333) reflection. To suppress the contamination, we replaced Si(111) with Si(511) in the present update [Fig. 1 ▸(*b*)], because Si(511) has the same lattice spacing with Si(333), allowing us to maintain the beamline geometry and experimental configurations without requiring extensive re-optimization of downstream components.

The crystal was fabricated with the dimensions 700 mm (length), 24 mm (width) and 20 mm (thickness) using mechanochemical surface polishing. A bending mechanism was employed for coarse horizontal focusing. This monochromator can theoretically generate higher-harmonic X-rays above 300 keV [*e.g.* from Si(15 3 3) reflections], although such energies are far beyond the usable range of the light source and the detector. In practice, the Si(511) reflection provides an essentially pure 113 keV X-ray beam with substantially reduced contamination from higher harmonics. This is supported by the scattering measurements, in which no diffraction features attributable to higher-harmonic components were observed.

The photon flux of the 113 keV monochromatic beam, measured by an ion chamber with Ar gas at the upstream end of the experimental hutch, was ∼1.2 × 10^10^ photons per second. This value was higher than that achieved with the previous Si(111) monochromator, because the latter configuration employed an attenuator to suppress the low-energy 37.8 keV component at a cost of reducing the beam intensity by around 30%.

### 2.2. High-throughput X-ray total scattering system at BL04B2

**Figure 2:**
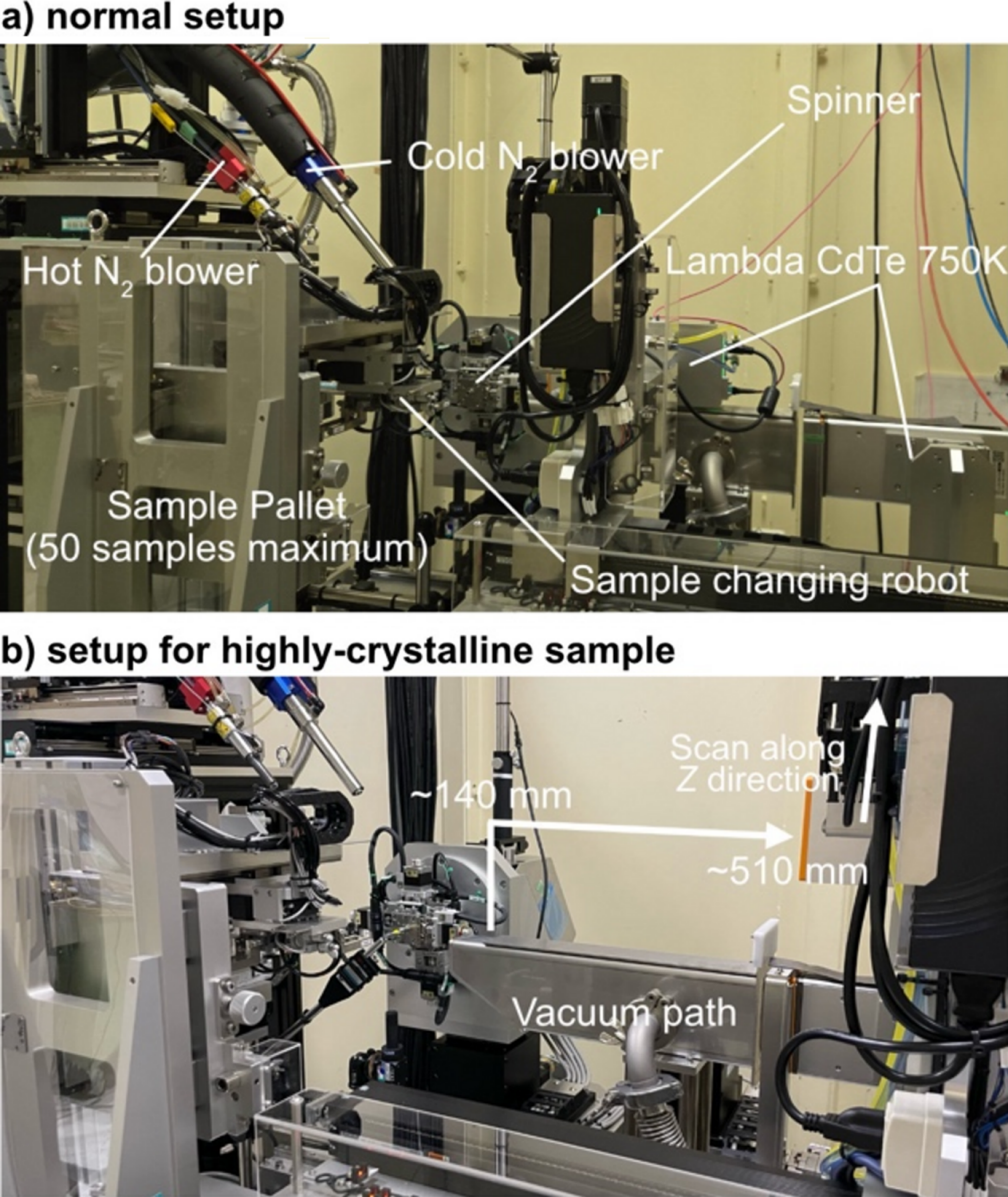
High-throughput X-ray total scattering setup and data reduction software installed at BL04B2. (*a*) Normal setup for X-ray total scattering and (*b*) setup for the highly crystalline sample.

Fig. 2 ▸(*a*) and Fig. S1 of the supporting information show the detailed configuration and components of the high-throughput X-ray total scattering diffractometer. The core optical elements – the four-blade slit, beam shutter and collimator – are precisely aligned on an optical bench. The incident X-ray beam is shaped by the slits to dimensions of 0.9 mm (vertical) and 1.5 mm (horizontal). After passing through the slits and collimator, the beam flux was measured at ∼3.4 × 10^9^ photons per second at 113 keV. The optical bench is mounted on a movable rail system that allows translation along the X-ray beam direction, which is crucial for accommodating various experimental setups around the sample position, such as high-pressure or hydro­thermal cells. The optical bench is positioned close to the sample to minimize air scattering during routine experiments, thereby improving the signal-to-noise ratio and enhancing the scattering data quality.

The high-throughput measurement mode and *in situ* measurement mode were used for measurements. The former was optimized for powder samples sealed in capillaries mounted on standardized sample holders placed into a pallet that could hold up to 50 samples simultaneously. The shape and interface of these sample holders and pallets were compatible with those utilized at other powder diffractometers in SPring-8 (Kawaguchi *et al.*, 2017 ▸). This facilitated seamless sample exchange across different experimental stations without requiring any remounting or repacking, achieving more efficient and easier operation as well as higher sample throughput during beam time. The sample holders were automatically manipulated by a sample-changing robot, which transferred each holder onto a motorized sample spinner. The position of each capillary was precisely aligned via optical image processing and multi-axis motor stage control, ensuring consistent beam alignment and measurement accuracy (detailed method is described in alignment method for a capillary in the supporting information). Sample exchange and alignment typically take 2–3 min. The measurement sequence is controlled by reading an Excel sheet that specifies the sample position on the pallet (up to 50 samples) and detailed measurement conditions such as exposure time and temperature.

Cryostream nitro­gen and hot-nitro­gen gas blowers precisely controlled the sample temperature within the ranges 100–473 K and 300–1100 K, respectively, to enable temperature-dependent measurements in this high-throughput configuration. The sample temperatures were calibrated using a K-type thermocouple inserted into dummy capillaries to ensure measurement accuracy during thermal cycling. As a result, the structural evolution in the sample could be characterized over a wide range of thermal conditions. This feature is particularly valuable for analyzing phase transitions, thermally activated processes and temperature-dependent disorders in amorphous and crystalline materials.

The sample spinner used in the high-throughput mode was retracted during *in situ* measurements, and a set of modular, multi-axis stages was introduced. These stages enabled the sample to independently move in three orthogonal directions with the travel ranges ±100 mm (*x* axis), ±50 mm (*y* axis) and ±25 mm (*z* axis), respectively. The setup can accommodate sample environments of approximately 200 mm along the beam direction and 300 mm perpendicular to the beam. In the vertical direction, the incident X-ray beam typically intersects the sample 70 mm above the standard flat mounting plate used at the beamline. Although the sample environment space was limited, this setup was suitable for installing compact reaction cells or environmental chambers. For example, a high-temperature furnace (TS-1500, Linkam Scientific Instruments) that controls temperature and atmospheric conditions (Kobayashi *et al.*, 2023 ▸) can be seamlessly integrated into the proposed system for conducting studies on materials under variable conditions such as reduction environment (Fig. S2). This *in situ* configuration also facilitated the real-time monitoring of chemical reactions, phase transformations and structural dynamics under non-ambient environments such as those with gas exposure or applied electric fields.

The system was equipped with two 2D CdTe photon-counting detectors (LAMBDA 750k, X-Spectrum GmbH) (Pennicard *et al.*, 2014 ▸) for high-energy X-ray total scattering experiments. They featured a fine pixel size of 55 µm and utilized 1 mm-thick CdTe sensors, offering high detection efficiency for high-energy photons (113 keV). The detector is typically positioned at a sample-to-detector distance of ∼510 mm, which corresponds to the accessible scattering-angle range 2θ = 0.2°–9.0° for this geometry. In this setup, the observed intensity at each pixel is typically below 500 counts per second. Therefore, the detector dead-time is sufficiently small and does not affect linearity, as confirmed by a previous report (Imai & Hatsui, 2024 ▸).

This setup could cover Bragg peaks at low angles (∼0.5°) from typical porous materials such as zeolites and metal–organic frameworks. To reduce background noise caused by air scattering in this configuration, a vacuum flight path was installed between the sample and detector. The length of this vacuum path is 370 mm along the X-ray direction and covers the scattering angle from 0 to 10°. The window material is polyimide film with the thickness of 25 µm. Using this setup, high-quality low-angle scattering data were collected for accurate PDF analysis.

The second detector captured X-ray scattering at 2θ = 8.5–30°. The sample-to-detector distance for this unit was tunable and was set to around 140 mm for amorphous or low-crystalline materials to maximize the *Q*-range and intensity. For measurements that require higher angular resolution, particularly for crystalline materials, this detector can also be positioned at 510 mm – the same distance used for the low-angle detector. A maximum scattering angle of 25° was achieved by adjusting its vertical (*z* axis) position, which was sufficient for most total scattering experiments. The experimental geometry could be flexibly adjusted by moving the detector based on the sample or the desired *Q*-range. Typical diffraction images captured by these detectors are shown in Fig. S3.

Both detectors were equipped with absorbers composed of silver (0.1 mm), molybdenum (0.1 mm) and copper (0.2 mm) plates. These were employed to suppress fluorescence that can considerably interfere with the accurate determination of PDFs. The beam stop was fabricated from lead and shielded with a 0.5 mm-thick tantalum plate to absorb secondary fluorescence generated by high-energy X-ray beams interacting with lead (Fig. S4). This beam stop is crucial for the current configuration because 113 keV X-rays can readily penetrate heavy metals and induce fluorescence, which may obscure weak total scattering signals. The bottom and back parts of the detector housing were protected by installing 2 mm-thick tungsten shielding components, which block backscattered high-energy radiation and suppress fluorescence from the beam stop. This shielding reduces parasitic radiation reaching the detector electronics and contributes to stable and reliable data collection (Zhou *et al.*, 2018 ▸). These careful shielding measures reduced systematic errors and enhanced the overall precision of the structural information derived from total scattering data.

The 2D images recorded by each detector are first azimuthally integrated using the *pyFAI* module (Kieffer *et al.*, 2020 ▸) to obtain 1D scattering curves. After data acquisition, background scattering is subtracted individually from each 1D dataset to isolate the sample scattering. The two 1D datasets are then merged by applying a scale factor to one dataset so that the intensities in the overlapping 2θ region match. Once the scaling is applied, the datasets are concatenated to form a continuous scattering curve. These data were saved as text files in a two-column format representing the scattering angle (2θ) versus intensity. This format is compatible with widely used data reduction software such as *PDFgetX3* (Juhás *et al.*, 2013 ▸), *GudrunX* (Soper & Barney, 2011 ▸) and other tools commonly used at SPring-8 (Ohara *et al.*, 2020 ▸; Tominaka *et al.*, 2018 ▸). This compatibility ensures that users can efficiently process and analyze the collected data using familiar workflows, facilitating rapid interpretation and dissemination of experimental results. Overall, the designed system offers high flexibility and reliability, providing robust support for a broad range of materials research applications.

A summary of the comparative performance between the new high-throughput system and the conventional setup is presented in Table S1of the supporting information, including parameters such as beam size, X-ray energy, photon flux, *Q* range and total measurement time. Owing to the difference in incident beam size, the total photon flux in the new system is approximately 1/3 of that in the conventional setup at 112.8 keV; nevertheless, the use of CdTe 2D detectors significantly shortens the measurement time. Furthermore, the new system is considerably faster than the conventional setup employing point-type detectors operated at 61 keV, demonstrating the validity and effectiveness of this upgrade.

## 3. Demonstration of typical measurements

### 3.1. High-energy X-ray total scattering measurements of silicate glass with different exposure times

X-ray total scattering experiments were performed on a silicate glass rod with a diameter of 1.0 mm at room temperature to evaluate the performance of the newly developed high-throughput measurement system installed at beamline BL04B2. The sample preparation and measurement conditions followed those reported in previous study (Yamada *et al.*, 2022 ▸) to enable comparability between the two systems.

**Figure 3:**
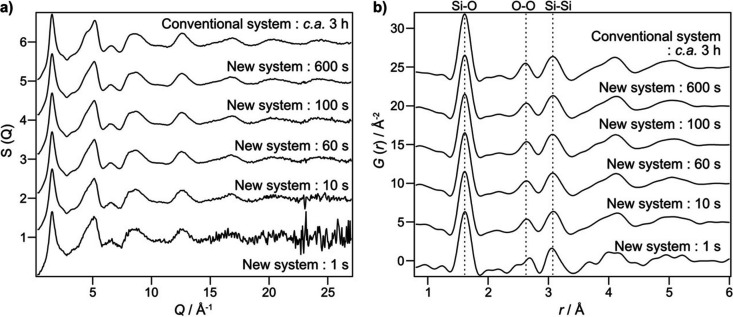
*S*(*Q*) and *G*(*r*) of silicate glass rod with a diameter of 1 mm determined by the new measurement system installed at BL04B2. (*a*) *S*(*Q*). (*b*) *G*(*r*). Data measured using the conventional system (with an exposure time of around 3 h) are also shown for reference.

Figs. 3 ▸(*a*) and 3 ▸(*b*) show the structure factor, *S*(*Q*), and the PDF, *G*(*r*), obtained at various exposure times of 1, 10, 60, 100 and 600 s, respectively, using the new high-throughput setup and those of 3 h measurement using the conventional system. Data with an exposure time of 1 s exhibited large noise; however, the quality of the *S*(*Q*) patterns considerably improved with increasing exposure time. The patterns collected with 60, 100 and 600 s exhibited sufficient signal-to-noise ratios to be utilized in atomistic modeling methods such as reverse Monte Carlo simulations (McGreevy, 2001 ▸) or to be compared with structural predictions from molecular dynamics simulations. Notably, the *S*(*Q*) pattern obtained using the conventional setup over 3 h had a comparable quality to that achieved with just 600 s of exposure time using the new system (Fig. S5). This clearly indicated that the high-throughput system enabled an order of magnitude faster data acquisition compared with the conventional setup at BL04B2.

Fig. 3 ▸(*b*) shows the advantages of the new system in terms of real-space structural analysis. Even in the data with a 1 s exposure time, characteristic *G*(*r*) peaks corresponding to the Si—O (∼1.6 Å), O–O (∼2.6 Å) and Si—Si (∼3.1 Å) pair correlations were observed. However, in the region beyond 3.5 Å, some artificial features arising from the noise in the *S*(*Q*) were observed. These artifacts were substantially reduced in the data obtained with an exposure time of 10 s and were almost completely suppressed in those obtained with exposure times of 100 s or longer. These findings indicate that an exposure time of at least 10 s is required to achieve reliable PDFs for the quantitative structural analysis of typical silicate glass samples.

The optimal exposure time may vary depending on the composition, atomic number and packing density of the samples. For most silicate-based glass materials, however, a 600 s exposure time is sufficient to obtain high-quality data across a wide range of interatomic distances. Such high efficiency implies that more than 100 total scattering measurements can be performed within a single day at this beamline, representing a substantial improvement in throughput and offering significant benefits for high-throughput structural studies of disordered materials.

### 3.2. Temperature-dependent measurements

**Figure 4:**
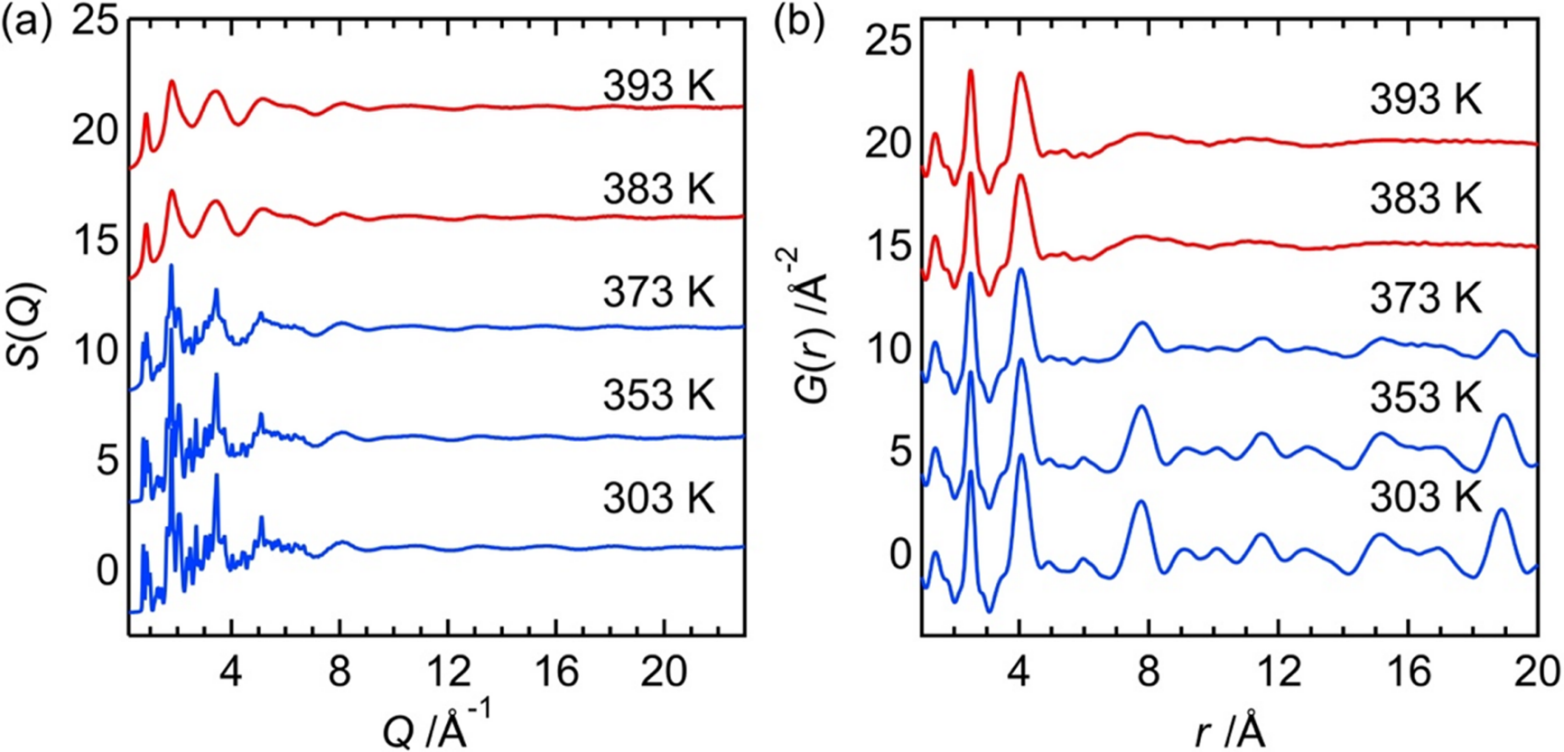
Temperature dependence of *S*(*Q*) and *G*(*r*) values for [C_5_H_9_N]_2_[MnBr_4_].

The ability of the newly developed system to perform temperature-dependent X-ray total scattering measurements was validated by employing it on an organic–inorganic hybrid compound, [C_5_H_9_N]_2_[MnBr_4_], that undergoes a solid–liquid phase transition (Shimono *et al.*, 2023 ▸). These findings are shown in Fig. 4 ▸.

Sharp Bragg peaks can be observed in the *S*(*Q*) at 303 and 353 K, indicating that the sample remained in the crystalline state [Fig. 4 ▸(*a*)]. Correspondingly, *G*(*r*) at these temperatures show well defined long-range correlations [Fig. 4 ▸(*b*)]. On heating the sample to 373 K, the intensity of Bragg peaks decreased and the long-range correlations (*e.g.* 7.9 and 19 Å) in *G*(*r*) were slightly reduced. This indicated a loss of long-range structural order, possibly due to enhanced thermal vibrations that weakened the cation–anion interactions. At temperatures above 383 K, the material transitioned to a liquid phase; this was confirmed by the appearance of a broad scattering pattern in the *S*(*Q*). However, local molecular structures were retained in the liquid state, as shown by the persistence of short-range features in the *G*(*r*) below 5 Å. These observations were consistent with previous *in situ* extended X-ray absorption fine structure measurements (Shimono *et al.*, 2023 ▸), confirming the structural integrity of molecular units even after melting.

Note that the conventional system performed these measurements in 1 day, whereas the new high-throughput system completed them in roughly 2 h, demonstrating a substantial improvement in measurement efficiency.

### 3.3. Highly crystalline materials

The high-throughput X-ray total scattering system yielded relatively low-resolution X-ray diffraction (XRD) patterns compared with other synchrotron XRD beamlines as a consequence of using high-energy X-rays. This indicated that the proposed system can be mainly used for determining the local structures of liquids, and amorphous and low-crystalline materials such as nanocrystals. Nevertheless, it can also be employed for evaluating highly crystalline materials when required, particularly for comparing the PDF patterns of samples with the same composition but different degrees of crystallinity under identical experimental conditions.

To this end, measurements were conducted for typical highly crystalline materials, namely CeO_2_ (NIST) and Ni. CeO_2_ and Ni powders were filled into borosilicate–glass capillaries with diameters of 0.5 and 1.0 mm, respectively.

**Figure 5:**
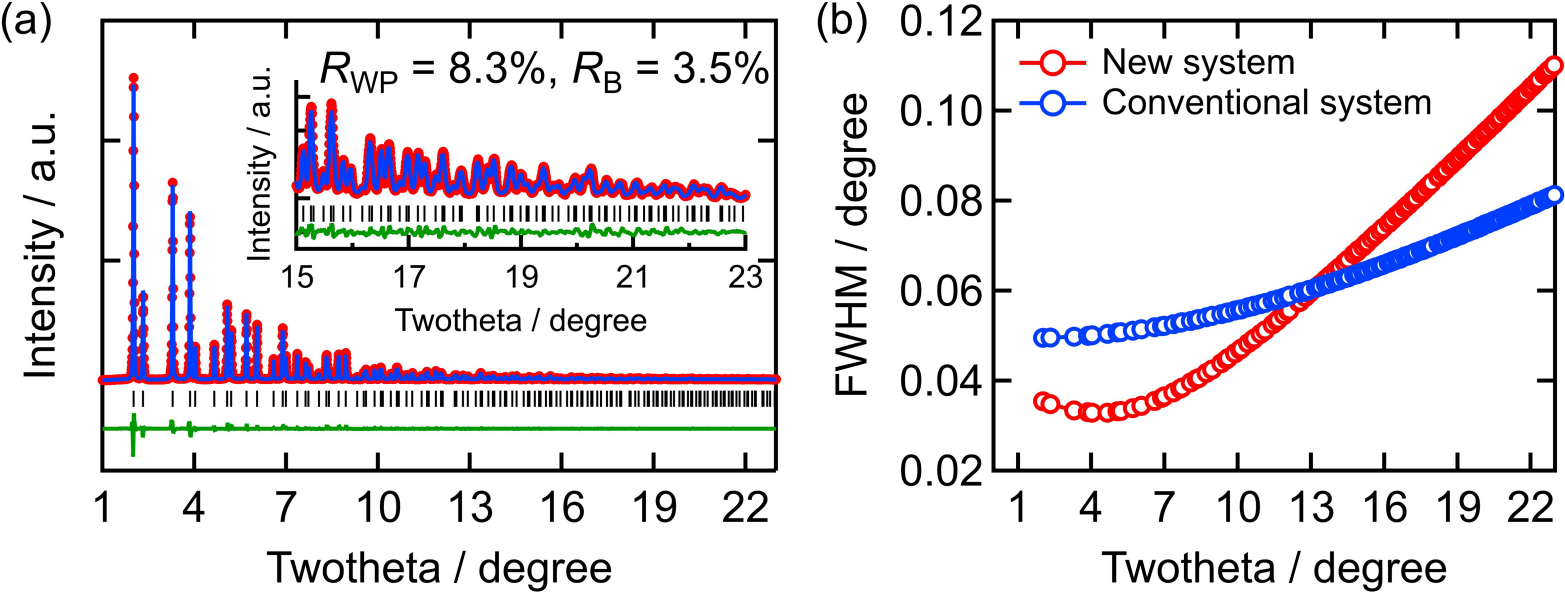
(*a*) XRD pattern and Rietveld refinement of CeO_2_. (*b*) Full width at half-maximum of Bragg peaks obtained via the Rietveld refinement of CeO_2_.

Fig. 5 ▸(*a*) shows the Rietveld refinement result of CeO_2_ with a 60 s exposure time using the *Jana2020* software (Petříček *et al.*, 2023 ▸). The red circles, blue lines, green lines and black vertical marks denote the experimental values, calculated values, difference curves and Bragg peak positions, respectively. As shown in this figure, the XRD pattern of CeO_2_ matches well with the simulation result (*R*_B_ = 3.5%), indicating that the current setup is also adequate for the analysis of crystalline materials. Because a wide azimuthal range is typically used at this beamline, the peaks at low scattering angles (1–5°) appear slightly broader due to the asymmetric shape of the incident beam.

The full width at half-maximum at low 2θ values below 10°, which is important for the analysis of crystalline structures, was narrower than that of XRD patterns obtained using the conventional setup [Fig. 5 ▸(*b*)]. This indicated that the developed system can be used for characterizing high-*Z* materials that require high-energy X-ray exposure or high-resolution XRD and PDFs.

**Figure 6:**
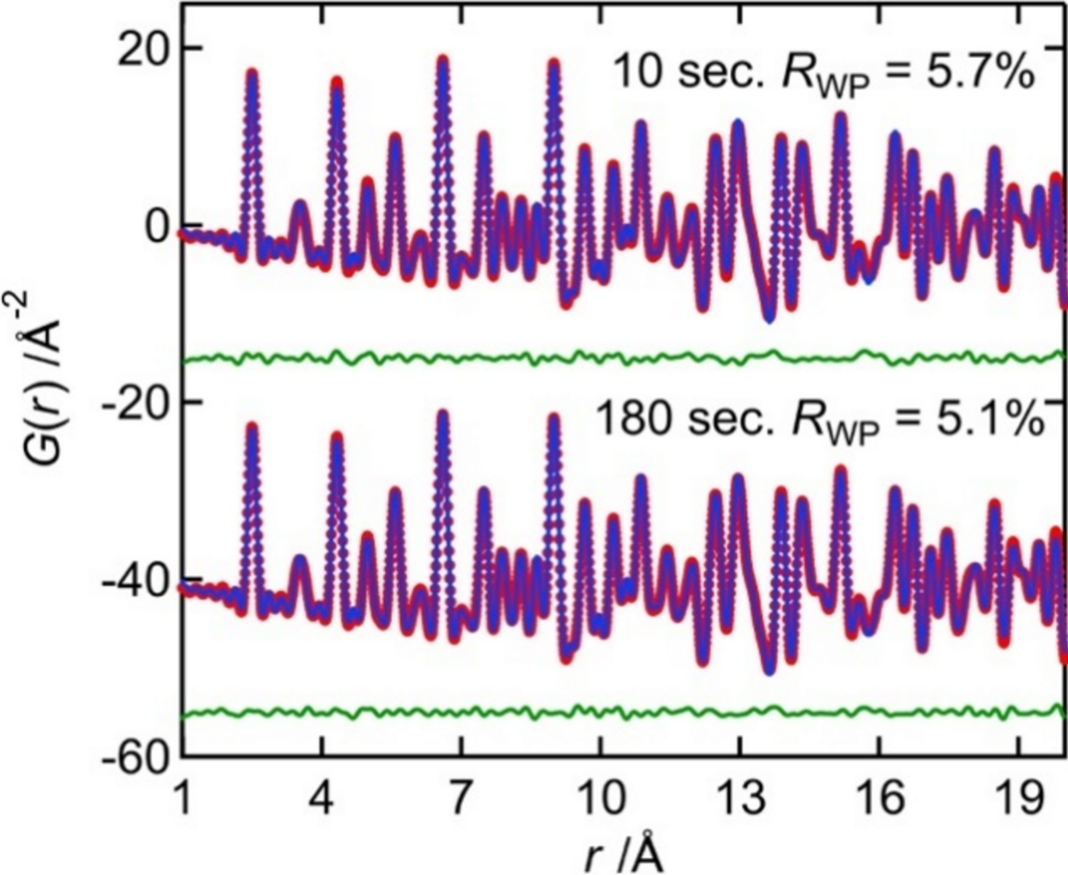
PDF fitting result for Ni obtained using *PDFgui*.

Fig. 6 ▸ shows the *G*(*r*) patterns and PDF refinement obtained using *PDFgui* (Farrow *et al.*, 2007 ▸) for Ni powder with exposure times of 10 and 180 s. The red circles, blue lines, and green lines represent the experimental values, calculated values and the difference curve, respectively. Both fitting patterns closely match the experimental PDF patterns obtained between 1 and 20 Å (*R*_WP_ = 5.1%), confirming the validity of the newly developed setup and its usefulness in evaluating highly crystalline materials. The *Q*_damp_ and *Q*_broad_ of new system obtained by this fitting are 0.025 and 0.017 respectively. These values are comparable to those of the conventional system (0.024 and 0.016).

From the results summarized in Figs. 5 ▸ and 6 ▸, it is evident that the X-ray total scattering data obtained using the new measurement system at BL04B2 possess sufficient accuracy for the quantitative analysis of highly crystalline materials.

## 4. Conclusions

A new system was installed at beamline BL04B2 in SPring-8 to reduce the measurement time of X-ray total scattering experiments. Before installing the diffractometer, the Si(111) monochromator in the optics hutch was replaced with the Si(511) monochromator to eliminate undesired effects from higher-harmonics X-rays. As a result, monochromatic X-rays with an energy of 113 keV were available at beamline BL04B2. The high-throughput X-ray total scattering diffractometer was installed in the downstream region of the experimental hutch. It comprised incident X-ray beam-shaping units, double 2D CdTe detectors, sample-changing robots, hot and cold nitro­gen blowers, and a sample-spinner stage. This system performed each X-ray total scattering measurement in ∼10 min, over ten times quicker than the time required using the conventional setup, enabling over 100 measurements a day. With this capability, BL04B2 in SPring-8 is now among the leading facilities worldwide for rapid X-ray total scattering measurements. This significant improvement will accelerate the evaluation of amorphous or nanocrystalline materials that require local distortion information and contribute to the establishment of new approaches that use data-driven methods such as materials science.

## Supplementary Material

Supporting Figues S1 to S5, Table S1. DOI: 10.1107/S1600577525011294/ye5075sup1.pdf

## Data Availability

The data used in this study are available from the corresponding author upon reasonable request.
