# Supplementary material for: High-throughput X-ray total scattering measurement system at BL04B2 of SPring-8
Source: J Synchrotron Radiat. 2026 Jan 22;33(Pt 2):516–22. doi: 10.1107/S1600577525011294 (PMC12948019; doi:10.1107/S1600577525011294)
Supplement: Supplementary file 1 [file s-33-00516-sup1.pdf]

JOURNAL OF SYNCHROTRON RADIATION

Volume 33 (2026)

Supporting information for article:

High-throughput X-ray total measurement system at BL04B2 of SPring-8

Hiroki Yamada, Seiya Shimono, Shogo Kawaguchi, Koji Ohara, Kei Watanabe, Michitaka Takemoto, Jo-chi Tseng, Masakuni Takahashi, Yuki Sada, Hiroshi Yamazaki, Haruhiko Ohashi, Yasuhiko Imai, Takaki Hatsui, Ichiro Inoue, Shigeru Kimura, Kunihisa Sugimoto, Makina Yabashi, Osami Sakata, Yuji Higo and Kenji Tamasaku

Alignment method for a capillary

The capillary alignment is performed using a CV5000 (Keyence) vision system, which allowed precise measurement of both the capillary orientation and its positional deviation during rotation. First, the capillary was observed at spinner angles of 0 and 180º. The holder was slightly rotated until the apparent sideways tilt between these opposite viewing angles was minimized, ensuring that the capillary remained upright with respect to the camera. The same procedure was then repeated at 90° and 270° to remove any forward–backward tilt, resulting in a capillary that was visually aligned along a straight axis when inspected from all orthogonal perspectives.

After the angular alignment, the capillary position was recorded at eight equally spaced angles over one full revolution. These measurements were fitted to determine the center of the circular trajectory traced by the capillary, which corresponds to the true mechanical rotation axis of the spinner. Finally, the capillary holder was translated laterally until the measured capillary center coincided with the calculated rotation axis. Through this procedure, the capillary was accurately centered and remained essentially stationary with respect to the X-ray beam during spinning, thereby ensuring stable and reproducible data acquisition.

Table. S1. Comparison of diffractometers of BL04B2 in SPring-8.

	New system (Si (511))	Conventional system (Si (511))	Conventional system (Si (220))
X-ray energy	112.8 keV	112.8 keV	61.3 keV
Typical beam size	0.9 mm (V)<br>1.5 mm (H)	1.5 mm (V)<br>2.0 mm (H)	1.5 mm (V)<br>2.0 mm (H)
Typical Q_{max}, dQ	27 Å^{-1}, 0.01 Å^{-1}<br>23 Å^{-1}, 0.003 Å^{-1}	24 Å^{-1}, 0.01 Å^{-1}	30 Å^{-1}, 0.01 Å^{-1}
Typical X-ray flux [photon/s]	3.4×10^9	1.2×10^{10}	2.0×10^{11}
Typical measurement time	5-10 min	180 min	120-180 min

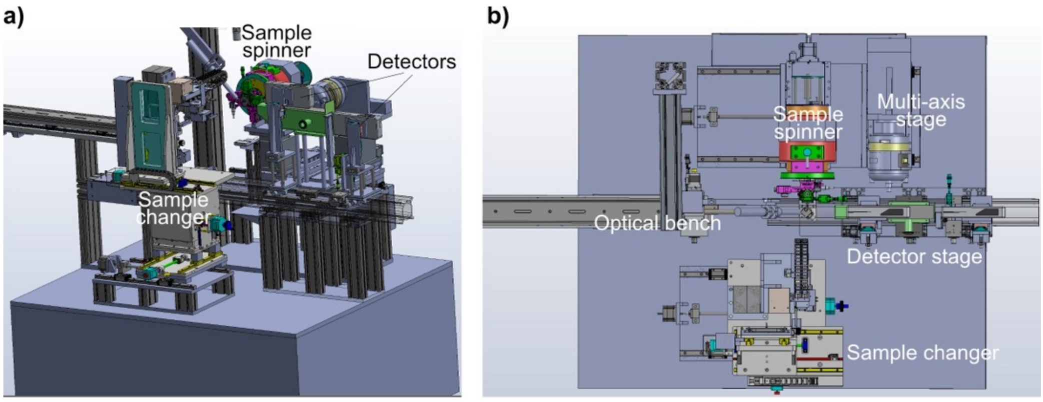

Figure 1 Schematic images of high-throughput X-ray total scattering system. a) side view. b) top view.

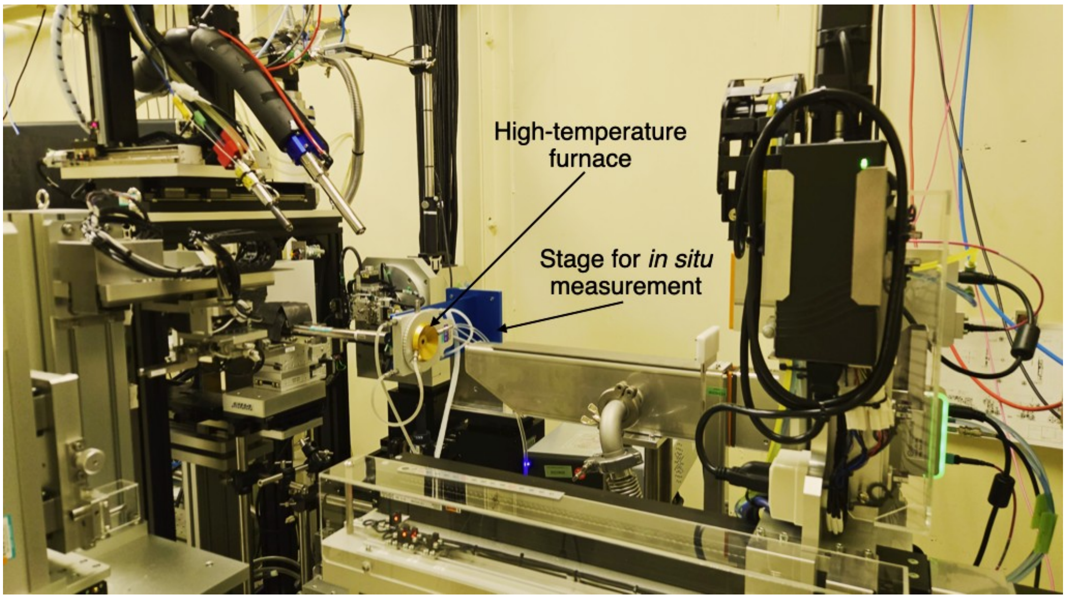

Figure 2 *In situ* measurement mode of high-throughput X-ray total scattering system. High-temperature furnace is installed in this photograph.

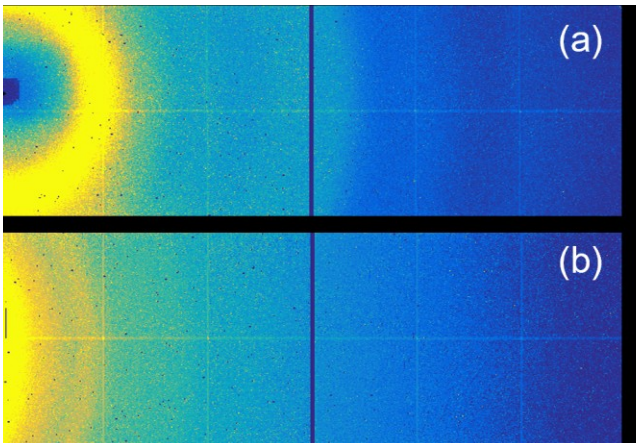

Figure 3 Typical two-dimensional diffraction images obtained with the present setup. The results for a silicate glass sample are shown as a representative example. (a) Image recorded by the detector covering the low-angle region. (b) Image recorded by the detector covering the high-angle region.

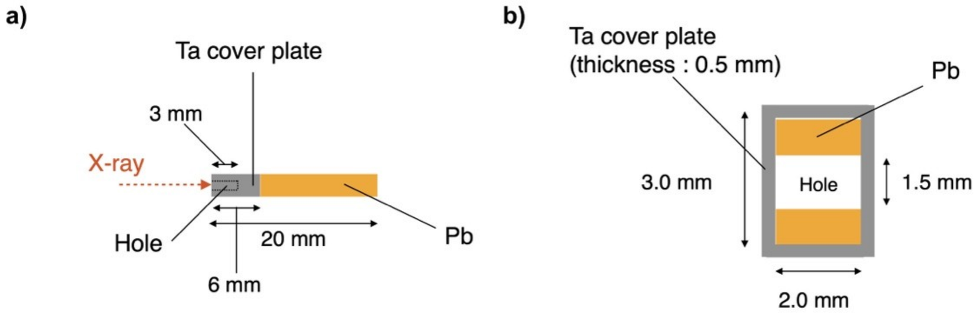

Figure 4 Schematic images of the beam stop used in this system. a) side view. b) front view. Noting that magnification of a and b is different for clarity.

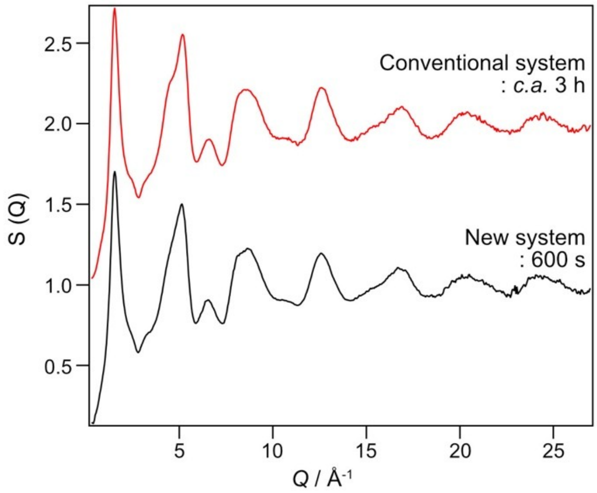

Figure 5 Comparison of S(Q) obtained using the new system with an exposure time of 600 s and the conventional system with an exposure time of 3 h.
